# Evaluating the quality of expert‐trained generative artificial intelligence when answering tracheostomy‐related questions

**DOI:** 10.1002/anr3.70065

**Published:** 2026-05-12

**Authors:** B. Gorst, E. Johnstone, J. Abbas, M. Moran, A. Payton, B. A. McGrath

**Affiliations:** ^1^ School of Biological Sciences, Faculty of Biology, Medicine and Health University of Manchester Manchester UK; ^2^ Division of Informatics, Imaging and Data Sciences University of Manchester Manchester UK; ^3^ Sentira XR St Albans Hertfordshire UK; ^4^ North West School of Anaesthetics Liverpool UK; ^5^ Manchester Academic Critical Care, Division of Infection, Immunity and Respiratory Medicine, School of Biological Sciences, Faculty of Biology, Medicine and Health University of Manchester, Manchester Academic Health Science Centre Manchester UK; ^6^ Department of Anaesthesia Manchester University NHS Foundation Trust Manchester UK

**Keywords:** artificial intelligence, reproducibility of results, tracheostomy

## Abstract

Tracheostomy training is effective, yet significant face‐to‐face training barriers remain. We aimed to develop, train and test a generative artificial intelligence instructor (AI‐Brendan). AI‐Brendan utilises the base model of ChatGPT‐4.0, augmented by a curated dataset of expert‐answered tracheostomy care questions, delivered with a contextually responsive conversational interface (Inworld). AI‐Brendan's responses to five randomly selected questions were compared with ChatGPT‐4.0 and five human expert responses using a modified Ensuring Quality Information for Patients tool, rated by six blinded independent adjudicators. A total of 450 Ensuring Quality Information for Patients scores were generated from responses (150 each from AI‐Brendan, ChatGPT‐4.0 and Experts), with no alarming responses. AI‐Brendan (mean score 12.6 ± 3.0) performed significantly better than ChatGPT‐4.0 (11.4 ± 2.4) and Experts (8.9 ± 3.6, ANOVA p < 0.01). Friedman testing demonstrated no significant differences across repeated responses, indicating consistency. Within‐question variability indicated highly consistent responses. Inter‐rater reliability was good (Cronbach's α = 0.863). Adjudicator's ability to correctly determine answer source was 50% for Experts, 39% for AI‐Brendan and 33% for ChatGPT‐4.0. Our expert‐trained generative artificial intelligence produced “better” answers than open‐source ChatGPT‐4.0 and experts when rated by the modified Ensuring Quality Information for Patients tool. A validated artificial intelligence instructor has significant implications for addressing training barriers, increasing efficacy, overcoming scheduling, financial or geographical limitations, offering consistent, accessible and flexible training.

## Introduction

A tracheostomy involves forming an artificial airway stoma in the neck by creating a connection between the skin and the anterior trachea. An indwelling tracheostomy tube is inserted to maintain patency. Whilst the classical indication for tracheostomy is actual or anticipated upper airway obstruction, the modern dominant indication is to facilitate recovery from invasive mechanical ventilation in the critically ill [1]. Tracheostomies and their safe management are a critical component of developed healthcare systems. An estimated 15,000–20,000 tracheostomies are performed annually in the UK, with published global estimates predicting around 250,000 new procedures per year in mature healthcare systems, and likely many more not quantified [2]. While tracheostomies offer several benefits, the nature of the artificial airway and associated physiological changes expose patients to risks. Several landmark papers have attempted to quantify risk, and literature consensus estimates that approximately 20–30% of tracheostomy patients will experience an adverse event during their hospital stay [3, 4]. Events can occur around the time of the procedure, or more commonly, in the post‐insertion period. Events can rapidly become life‐threatening, especially if the patient requires oxygen or is ventilator dependent. Patient‐focused studies have highlighted that in addition to harm, anxiety, depression and inability to vocalise, eat or drink effectively are prevalent [5].

A variety of local, national and international quality improvement studies have demonstrated that care can be delivered more safely and effectively whilst improving the patient experience [1, 2, 4, 5]. Any healthcare worker who may encounter a patient with a tracheostomy should be trained to a minimum standard to contribute to high‐quality care delivered within safe systems. The variety, volume and turnover of multidisciplinary staff who are required to ensure optimal care means that delivering tailored education is challenging. The rising costs for high and low‐fidelity simulation manikins, equipment and suitable training venues restrict the scalability of training. Furthermore, the requirement for expert teachers is under constant tension when balanced against the service commitments of trainers and trainees, often with limited study opportunities and budgets, and competing demands for training.

The UK National Tracheostomy Safety Project (NTSP) has attempted to standardise multidisciplinary training programmes by providing freely available resources and training packages for staff, patients and families (www.tracheostomy.org.uk). Training staff using NTSP resources has been shown to improve metrics of quality, safety, organisational efficiency and healthcare economics [5, 6]. The pandemic drove many educational activities on‐line and the NTSP developed a virtual reality version of their half‐day training, demonstrating that it could be delivered remotely and effectively, achieving learning outcomes and knowledge retention equivalent to face‐to‐face training [7]. However, these courses still required expert instructors to plan and deliver training. Our aim was to explore the role of generative artificial intelligence (AI) in teaching evidence‐based tracheostomy care. Artificial intelligence is commonly understood as the capacity of machines to undertake functions traditionally dependent on human cognitive abilities, with the promise of increasing efficiency and reducing costs when applied to healthcare [8]. Large language models (LLM), such as ChatGPT from OpenAI, use ‘transformer model’ neural network architecture to process vast quantities of data. Healthcare publications relating to ChatGPT increased 10‐fold over a 3‐month period in 2025 [9], with imaging reporting and patient information uses particularly prevalent. However, healthcare AI is still in its infancy, with challenges remaining to ensure the accuracy and reliability of information provided. This is particularly important as medical education requires appropriate precision and rigour without which patient safety may be put at risk [10].

This study aims to develop, train and test a generative AI which could support tracheostomy education. Our primary objective was to determine whether a trained AI‐instructor (AI‐Brendan) performed better than ChatGPT‐4.0 or a panel of expert clinicians in answering tracheostomy care questions, when responses were rated independently using a validated tool. Secondary objectives include assessing conciseness of answers and the ability of healthcare staff to correctly identify the source of responses.

## Methods

AI‐Brendan is a domain‐optimised curated interactive AI‐avatar, based on one of the authors with a recognisable profile in the field of tracheotomy safety (Supporting Information, Figure S1). Incorporating personality traits into AI enhances user engagement and fosters trustworthiness [11]. AI‐Brendan utilises ChatGPT‐4.0 base model, augmented by an additional curated dataset assembled from 905 tracheostomy care questions asked and captured during NTSP training courses between 2012 and 2023. These questions had been previously answered by course teaching faculty independently of this study. SentiraXR (Manchester, UK) developed the avatar using the Unity Engine (Unity Technologies, San Francisco, US) with an Inworld AI integration (Inworld, Mountain View, US) to enable contextually responsive and realistic conversations. Voice cloning software (ElevenLabs, London, UK) was used for high‐fidelity voice synthesis. Avatar creation and animation were completed using Character Creator‐4 and iClone‐8 (Reallusion, Taiwan). A cloud‐based streaming environment enhances accessibility by allowing the software to be run remotely from multiple devices including tablets, phones and computers. By embodying the specialised knowledge base within a human‐like avatar, AI‐Brendan aims to create an engaging and accessible learning experience.

Ethical approval was not required (Health Research Authority decision tool). A public‐facing tracheostomy care survey (Qualtrics‐QM) was launched at www.tracheostomy.org.uk on 01/02/2025. From 116 responses submitted by 01/05/2025, 45 distinct questions were identified. Five questions were selected using a random sequence generated in R Studio (2025.09.1 + 401) (Supporting Information, Table S1). Selected questions were then asked of the three groups: ChatGPT‐4.0; AI‐Brendan; a group of five experts (experienced multidisciplinary trainers with a minimum of 5 years of clinical experience in tracheostomy care). To account for potential variability in generative AI outputs, each LLM was challenged verbatim without changing phrasing, punctuation or formatting on five separate occasions. This generated 25 free‐text answers per group and a total of 450 responses which were individually scored by adjudicators (Supporting Information, Figure S2).

Answers were assessed using the EQuIP tool, validated as a reliable and objective measure with strong inter‐rater reliability across healthcare contexts, including generative AI content [12]. This framework provides a systematic method for evaluating three main domains: content, data identification and structure. The original EQuIP tool consists of 36 items with four possible responses to questions: yes, partly yes, no and not applicable. We adapted the tool by removing questions not related to our methods (internet search engine‐related) and employed a binary yes/no (score 1 or 0) used in several other studies [12, 13]. The 24‐item modified tool is detailed in Supporting Information (Table S2). The five clinical experts provided independent free‐text answers, without access to external resources, search engines, AI chat resources or textbooks, with response time limited to 2 min. Response lengths were suggested at 1–3 sentences from both experts (by request) and ChatGPT‐4.0 (by configuration).

Six independent, blinded adjudicators assessed the answers presented in an anonymised Qualtrics survey instrument in random order (R Studio), generating a score from 0 to 25 for each response. Adjudicators were not tracheostomy subject experts (university students undertaking a variety of courses). Adjudicators received standardised training consisting of a PowerPoint presentation explaining the study, the modified EQuIP tool, example answers and example scores. After scoring, one of the authors (BAM) reviewed all answers to judge accuracy and identify any critical content errors from a clinical perspective. Data distributions were examined in SPSS 29.0 (IBM Corp, USA) and mean (standard deviation) or median (inter‐quartile range) were reported, as appropriate. One‐way Welch Analysis of Variations (ANOVA) tests were used to identify group differences, described by mean differences and 95% confidence intervals (95%CI).

Differences in EQuIP scores across the five repeated responses to the same question were assessed using the Friedman test for related samples, while within‐question variability in response quality was quantified using the coefficient of variation (standard deviation divided by the mean), with lower values indicating greater consistency [14]. Conciseness of responses (mean word count) between each group of responders was also evaluated. Intraclass Correlation Coefficient measured response reliability and Cronbach's Alpha assessed inter‐rater agreement.

## Results

**Figure 1:**
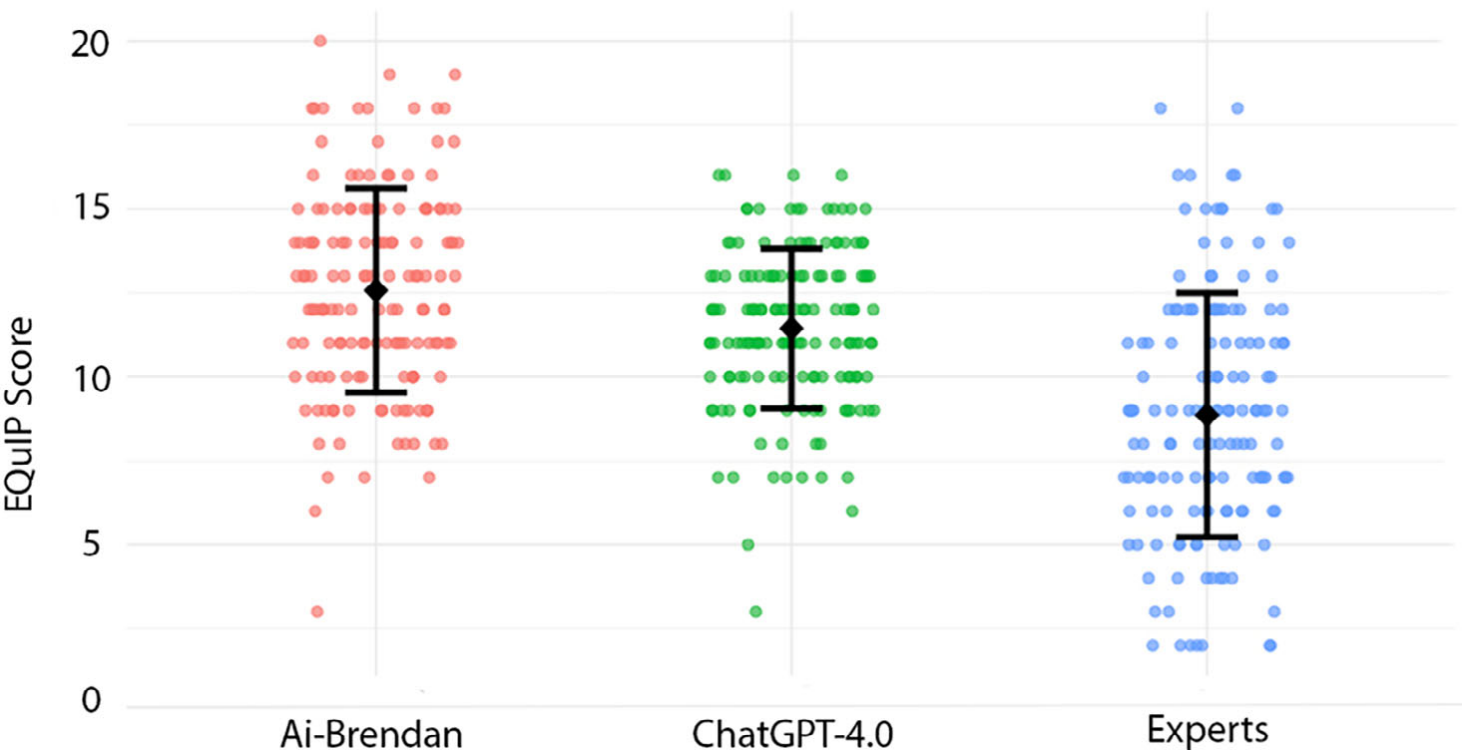
Modified Ensuring Quality Information for Patients tool scores grouped by responder group. Raw data points are shown with mean (black dot) and standard deviation (bars). Group difference p < 0.01.

A total of 450 EQuIP scores were generated from the responses (150 each from AI‐Brendan, ChatGPT‐4.0 and the experts). Reassuringly, no incorrect or alarming responses were generated, and all responses were considered appropriate. Mean EQuIP scores progressively increased from the experts (8.9), through ChatGPT‐4.0 (11.4) to AI‐Brendan (12.6, p < 0.01, Table 1). Post hoc analysis demonstrated significant group differences: AI‐Brendan was a mean difference of 1.1 (95%CI 0.4–1.9, p = 0.001) EQuIP points higher than ChatGPT‐4.0 and 3.7 (2.8–4.6, p < 0.001) higher than the expert panel (Fig. 1). AI‐Brendan generated a significantly greater mean word count than both ChatGPT‐4.0 (mean difference 16 whole words) and the experts (17 words, both p < 0.001, Table 1).

**Table 1:** Modified Ensuring Quality Information for Patients (EQuIP) tool score and answer word counts by responder group.

	Answer source						Group difference
	AI‐Brendan		ChatGPT‐4.0		Experts		
	Mean	(SD)	Mean	(SD)	Mean	(SD)	p
EQuIP score	12.6	(3.0)	11.4	(2.4)	8.9	(3.6)	<0.01
Answer word count	49.8	(14.6)	34.0	(4.1)	33.0	(18.4)	<0.001

	FTS	p	FTS	p	FTS	p
Friedman test	4.63	0.33	5.62	0.23	5.76	0.22

FTS, Friedman test statistic.

Friedman testing showed no statistically significant differences in EQuIP scores across repeated responses for AI‐Brendan, ChatGPT‐4.0 and experts (all p > 0.05, Table 1), indicating consistency in response quality (EQuIP scores). Within‐question variability was summarised using the coefficient of variation. Pooled mean values were 0.10 and 0.09 for AI‐Brendan and ChatGPT‐4.0, respectively, considered as indicating very low variability across repeated answers (Supporting Information, Table S3). Experts had a pooled mean value of 0.25, considered as indicating moderate variability, with noticeable differences between responses.

Inter‐rater reliability was considered good (Cronbach's α = 0.863) among the six independent adjudicators. Intraclass Correlation Coefficients demonstrated moderate inter‐rater consistency for individual adjudicators (0.5 [95%CI 0.4, 0.6], p < 0.001) and good inter‐rater consistency when averaging the six adjudicators' scores (0.9 [0.8–0.9], p < 0.001). Finally, the adjudicators' ability to correctly determine the source of the answers was experts (50%), AI‐Brendan (39%) and ChatGPT‐4.0 (33%).

## Discussion

Our comparative analysis has demonstrated that an expert‐trained generative LLM (AI‐Brendan) generates “better” answers than open‐source LLM (ChatGPT‐4.0) and human tracheostomy experts when rated by the modified EQuIP tool. Whilst AI‐Brendan's responses were significantly longer than the other groups, this did not negatively impact EQuIP scores. Raters were not able to differentiate easily between the source of answers. A validated AI instructor has significant implications for addressing the barriers in face‐to‐face education and training. It has the potential to increase efficacy by providing on‐demand training, overcoming the limitations posed by scheduling or geographical restrictions, and offering consistent, accessible and flexible training which is difficult to deliver with human trainers.

Both AI‐Brendan and ChatGPT‐4.0 scored significantly higher than experts, reflecting growing AI capabilities. The human‐trained AI‐Brendan reassuringly outperformed ChatGPT‐4.0. This reflects optimal practice in curating an LLM with strong human‐generated input. The EQuIP tool performed consistently well with good fidelity, indicating that the adjudicators were able to apply the metric consistently, despite their independence. Half of the raters were medical students and half from humanities courses, highlighting that responses by AI‐Brendan were understandable by a non‐specialist audience.

Optimal response length represents a balance of content, depth of understanding and clarity. Variations may represent adaptations to the specific context and demands of the prompt [15]. Experts provided the most concise responses, but with the most inconsistency. The probable cause of AI‐Brendan producing lengthier responses may be the absence of prompting restrictions. It is plausible that the higher mean EQuIP score of LLMs was driven by their higher word counts, although the scoring domains do not obviously reinforce this. As expected, our results demonstrated that human expert responses were correctly identified 50% of the time, whereas AI sources were less frequently identified. Subtle syntax or conversational phrasing remained in the human responses which likely accounts for this result. Other investigators have identified variable success rates ranging from 24 to 70% for AI‐generated text and up to 63% for human text [16].

A similar study found ChatGPT‐4.0 to be superior to nurses in answering tracheostomy care questions [17]. This study focused purely on accuracy, with adjudicators rating each response as ‘correct’, ‘partially correct’ or ‘incorrect’. This approach falls short of the holistic evaluation required to assess an educational tool intended for communication. Conversational AI‐avatars have been recognised for enhancing user engagement and creating immersive learning. Although research has investigated the use of AI‐avatars in healthcare, a gap persists in studies assessing the quantifiable quality of responses from AI‐avatars specifically designed as conversational knowledge bases. Our research provides a contribution to the academic landscape by showing that AI‐avatars can deliver high‐quality information.

Our study has several limitations. The sample size of five question prompts is limited, although the content was drawn from real‐world questions and selected at random. Additionally, only standalone responses were assessed, without any subsequent interactions, since the dialogue was reset between inquiries for the LLM. While suitable for assessing objective responses, this does not accurately reflect the full complexity of an educational environment, where follow‐up questions, requests for clarification and adaptive guidance may be anticipated. The scope of the study did not assess the attitudes of users towards AI‐Brendan. Finally, a further limitation is the rapidly evolving landscape of large language models. While ChatGPT‐4.0 was used as the comparator, newer models, such as ChatGPT‐5.2, Claude and Gemini, were not evaluated and future studies should include these systems. This study focused on the quality of its responses; in practice, user trust, ease of use and ethical concerns are integral elements for successful adoption.

## Conclusion

Our findings demonstrate that a trained generative AI produced superior quality responses to real‐world questions about tracheostomy care than an untrained LLM and human experts. Reassuringly, no incorrect or alarming answers were generated. This study provides evidence that specialised AI‐avatar LLM can generate high‐quality answers to medical inquiries and may be a valuable companion resource for patients, families and healthcare staff working in specialist areas where the opportunity to ask questions of human staff may be limited. Further research should evaluate the ability of AI‐Brendan to perform in a conversational dynamic environment and include the user attitudes of intended audiences.

## Supporting information


**Figure S1.** The AI‐Brendan avatar interface.
**Figure S2.** Study methodology flow chart.
**Table S1.** Test questions selected.
**Table S2.** Question domains for the modified EQuIP score used.
**Table S3.** Within‐question variability summarised using the coefficient of variation.
