# Supplementary material for: Evaluating the quality of expert‐trained generative artificial intelligence when answering tracheostomy‐related questions
Source: Anaesth Rep. 2026 May 12;14(1):e70065. doi: 10.1002/anr3.70065 (PMC13163142; doi:10.1002/anr3.70065)
Supplement: Supplementary file 1 — Figure S1. The AI‐Brendan avatar interface. Figure S2. Study methodology flow chart. Table S1. Test questions selected. Table S2. Question domains for the modified EQuIP score used. Table S3. Within‐question variability summarised using the coefficient of variation. [file ANR3-14-e70065-s001.docx]

**Supplementary Material**

**Table S1.** Test Questions selected.

| **Test questions** |
| --- |
| 1. What are the risks associated with tracheostomy insertion? |
| 1. Who should be part of the tracheostomy multidisciplinary team? |
| 1. Is a surgical tracheostomy better than a percutaneous tracheostomy? |
| 1. How can we help patients speak after tracheostomy in the intensive care unit? |
| 1. Where is the best place to start when trying to improve care for tracheostomy patients in a hospital? |

**Table S2.** Question domains for the modified EQuIP score used. Answers were scored as Yes (1 point) or No (0 points).

| Content QUESTIONS | |
| --- | --- |
|  | The answer provides a… |
| Q1 | Description of tracheostomy problem / incident / treatment |
| Q2 | Definition of the purpose of tracheostomy treatment or intervention |
| Q3 | Description of alternative options, advice or management |
| Q4 | Description of the sequence of the intervention or guidance |
| Q5 | Description of qualitative benefits for the patient |
| Q6 | Description of quantitative benefits for the patient |
| Q7 | Description of qualitative risks and complications |
| Q8 | Description of quantitative risks and complications |
| Q9 | Content addressing quality of life issues |
| Q10 | Description of how potential complications or side effects will be dealt with |
| Q11 | Description of precautions that the patient may take |
| Q12 | Mention of alert signs or symptoms that patients, staff or carers may detect |
| Q13 | Set of specific details of other sources of reliable information/support |
| Q14 | Coverage of all relevant parts on the topic |
| Identification of data QUESTIONS | |
| Q15 | The answer refers to source of information or evidence base |
| Structure QUESTIONS | |
|  | The answer provides… |
| Q16 | Use of everyday language, explains complex words or jargon |
| Q17 | Use of generic names for all medications or products |
| Q18 | Use of short sentences (<15 words on average) |
| Q19 | A document which personally addresses the reader |
| Q20 | A tone is respectful |
| Q21 | Information is clear (no ambiguities or contradictions) |
| Q22 | Information is balanced |
| Q23 | Information is presented in a logical order |
| Q24 | A design and layout which are satisfactory |

**Table S3.** Within-question variability summarised using the coefficient of variation. Values ≤ 0.10 are considered as indicating very low variability (highly consistent responses) across repeated responses. Values of 0.2-0.3 are considered as indicating moderate variability, with noticeable differences between responses [17].

| Responder | Question | Mean EQuIP score | Standard deviation | Range of EQuIP scores | Coefficient of variation | Pooled mean coefficient of variation |
| --- | --- | --- | --- | --- | --- | --- |
| AI-Brendan | 1 | 12.60 | 1.62 | 4.33 | 0.13 | 0.10 |
| AI-Brendan | 2 | 9.40 | 0.84 | 2.00 | 0.09 |  |
| AI-Brendan | 3 | 14.77 | 0.92 | 2.33 | 0.06 |  |
| AI-Brendan | 4 | 13.03 | 1.06 | 2.17 | 0.08 |  |
| AI-Brendan | 5 | 13.07 | 1.93 | 4.50 | 0.15 |  |
| ChatGPT | 1 | 12.43 | 0.76 | 1.83 | 0.06 | 0.09 |
| ChatGPT | 2 | 11.10 | 0.68 | 1.83 | 0.06 |  |
| ChatGPT | 3 | 10.53 | 1.20 | 3.00 | 0.11 |  |
| ChatGPT | 4 | 12.07 | 1.30 | 3.17 | 0.11 |  |
| ChatGPT | 5 | 11.07 | 1.22 | 2.83 | 0.11 |  |
| Experts | 1 | 7.50 | 1.52 | 4.17 | 0.20 | 0.25 |
| Experts | 2 | 6.40 | 1.53 | 3.17 | 0.24 |  |
| Experts | 3 | 11.37 | 3.15 | 7.83 | 0.28 |  |
| Experts | 4 | 9.90 | 1.80 | 4.33 | 0.18 |  |
| Experts | 5 | 9.13 | 3.11 | 7.83 | 0.34 |  |

**Figure S1.** The AI-Brendan avatar interface.


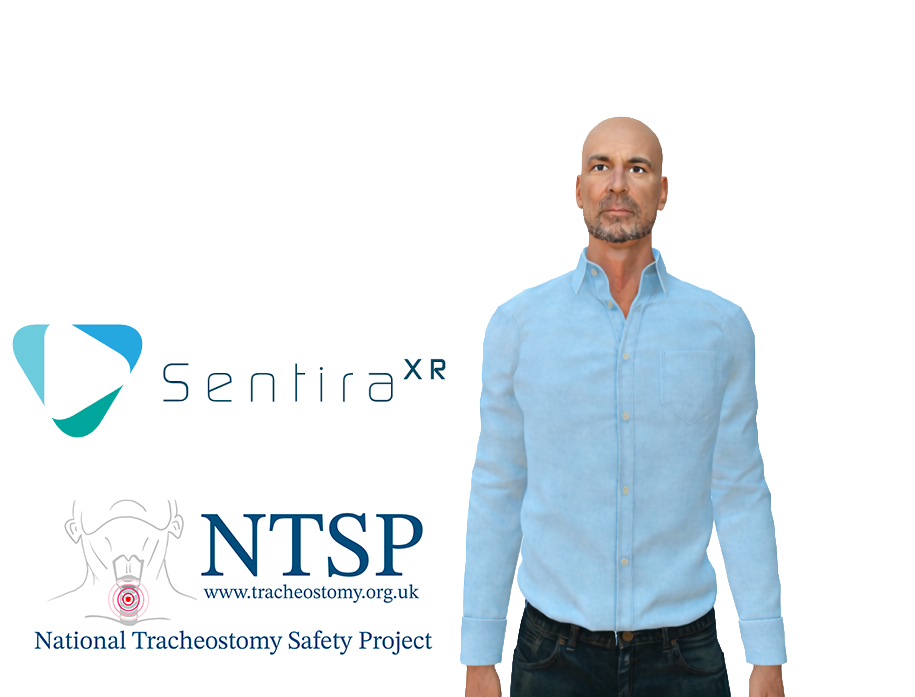


**Figure S2.** Study methodology flow chart.

**
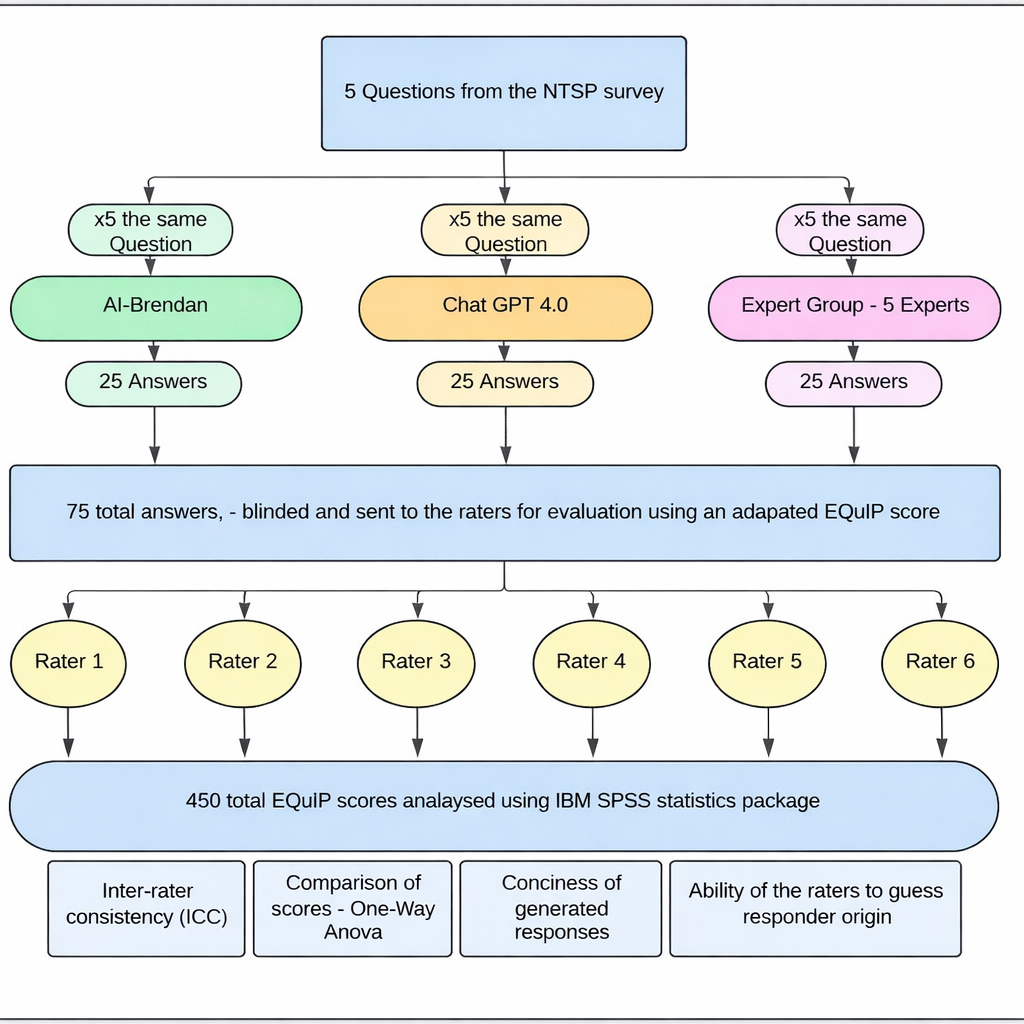
**
